# Progress towards Bait Station Integration into Oral Rabies Vaccination Programs in the United States: Field Trials in Massachusetts and Florida

**DOI:** 10.3390/tropicalmed2030040

**Published:** 2017-08-21

**Authors:** Brian M. Bjorklund, Betsy S. Haley, Ryan J. Bevilacqua, Monte D. Chandler, Anthony G. Duffiney, Karl W. von Hone, Dennis Slate, Richard B. Chipman, Ashlee Martin, Timothy P. Algeo

**Affiliations:** 1 United States Department of Agriculture, Animal and Plant Health Inspection Service, Wildlife Services, 9 Main St., Suite 1M, Sutton, MA 01590, USA; ryan.bevilacqua@aphis.usda.gov; 2 United States Department of Agriculture, Animal and Plant Health Inspection Service, Wildlife Services, National Rabies Management Program, 59 Chenell Dr., Suite 2, Concord, NH 03301, USA; betsy.s.haley@aphis.usda.gov (B.S.H.); dennis.slate@aphis.usda.gov (D.S.); richard.b.chipman@aphis.usda.gov (R.B.C.); ashlee.d.martin@aphis.usda.gov (A.M.); 3 United States Department of Agriculture, Animal and Plant Health Inspection Service, Wildlife Services, 463 West St., Amherst, MA 01002, USA; monte.d.chandler@aphis.usda.gov; 4 United States Department of Agriculture, Animal and Plant Health Inspection Service, Wildlife Services, 2803 Jolly Rd., Suite 100, Okemos, MI 48864, USA; anthony.g.duffiney@aphis.usda.gov; 5 Yarmouth Division of National Resources, 424 Route 28, West Yarmouth, MA 02673, USA; kvonhone@yarmouth.ma.us; 6 United States Department of Agriculture, Animal and Plant Health Inspection Service, Wildlife Services, 59 Chenell Dr., Suite 7, Concord, NH 03301, USA; timothy.p.algeo@aphis.usda.gov

**Keywords:** bait stations, oral rabies vaccination, raccoon, rabies, vaccine, non-target

## Abstract

Bait stations for distribution of oral rabies vaccine baits are designed for rabies management in highly-developed areas where traditional distribution of oral rabies vaccine baits may be difficult. As part of national efforts to contain and eliminate the raccoon (*Procyon lotor*) variant of the rabies virus (raccoon rabies) in the eastern United States, the United States Department of Agriculture, Animal and Plant Health Inspection Service, Wildlife Services program, distributed vaccine baits by bait stations experimentally and operationally in Massachusetts during 2006-present, and in Florida during 2009–2015. In Massachusetts, a rabies virus-neutralizing antibody (RVNA) response of 42.1% for raccoons captured in areas baited with high density bait stations during 2011–2015 was achieved, compared with 46.2% in areas baited by hand, suggesting the continuation of this as a strategy for the oral rabies vaccination (ORV) program there, and for similar locations. Non-target competition for vaccine baits is problematic, regardless of distribution method. In Massachusetts, bait station visitation rates for targeted raccoons and non-target opossums (*Didelphis virginiana*) were similar (1.18:1) during 2006–2009 (*p* > 0.05). Bait station modifications for reducing non-target uptake were tested, and in Massachusetts, reduced non-target bait access was achieved with two design alternatives (*p* < 0.001). However, no difference was noted between the control and these two alternative designs in Florida. Due to ongoing trials of new vaccines and baits, the bait station performance of an adenovirus rabies glycoprotein recombinant vaccine bait, ONRAB^®^ bait (Artemis Technologies, Guelph, ON, Canada) and a vaccinia-rabies glycoprotein recombinant vaccine bait, RABORAL V-RG^®^bait (Merial Limited, Athens, GA, USA), was compared. While uptake of the ONRAB bait was greater in Massachusetts (*p* < 0.001) in this limited trial, both types performed equally well in Florida. Since bait station tampering or theft as well as potential human bait contacts has been problematic, performance of camouflaged versus unpainted white bait stations was analyzed in terms of internal temperatures and maintaining a stable bait storage environment. In Massachusetts, camouflaged bait station interiors did not reach higher average temperatures than plain white bait stations in partially- or fully-shaded locations, while in Florida, camouflaged bait stations were significantly warmer in light exposure categories (*p* < 0.05). As ORV operations expand into more heavily-urbanized areas, bait stations will be increasingly important for vaccine bait distribution, and continued refinements in the strategy will be key to that success.

## 1. Introduction

Oral rabies vaccination (ORV) is an effective and socially acceptable strategy for wildlife rabies management [1]. Various ORV strategies have been employed successfully, including for the control of fox rabies in Western Europe [2,3] and Canada [4,5,6]. ORV has been used in Texas to eliminate canine rabies in coyotes (*Canis latrans*) [7,8], and to eliminate and prevent rabies in gray foxes (*Urocyon cinereoargenteus*) [9].

The United States Department of Agriculture, Animal and Plant Health Inspection Service, Wildlife Services (WS), National Rabies Management Program (NRMP), is conducting cooperative ORV operations to prevent the westward spread of the raccoon (*Procyon lotor*) variant of the rabies virus (raccoon rabies) into the mid-western states and eastern Canada (Phase I), and has begun work towards its eventual elimination from the eastern United States (Phase II) [1]. Much of the eastern United States is highly developed and features high human densities. Traditional aerial ORV strategies are complicated in these areas by high off-times of vaccine bait distribution equipment due to treatment area development, and concern for potential human and pet contact with vaccine baits. Bait stations for distribution of oral rabies vaccine baits provide an opportunity to conduct ORV plans at minimized risk levels in settings such as these.

**Figure 1:**
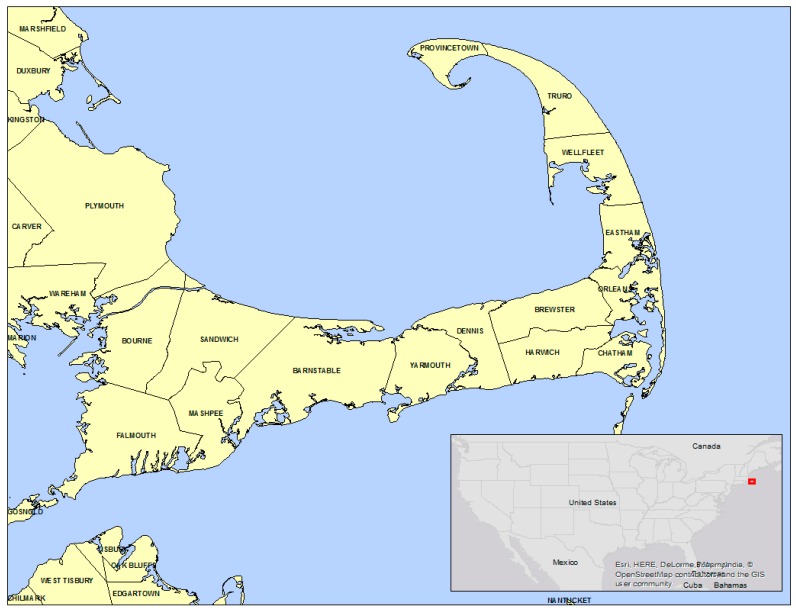
Cape Cod, Massachusetts, USA.

In 2001, WS began full-time collaboration on the Cape Cod Oral Rabies Vaccination Program (CCORV) with Tufts University and other cooperators as part of national wildlife rabies control efforts (Figure 1).

The primary objective of the CCORV was to use ORV in tandem with the physical barrier of the Cape Cod Canal to prevent the spread of raccoon rabies onto peninsular Cape Cod. In 2003, after an increase in raccoon rabies cases within the CCORV zone, vaccine bait distribution efforts were modified to reduce the risk of raccoon rabies spreading onto peninsular Cape Cod. Despite this, raccoon rabies was detected for the first time there in March 2004. A WS trap-vaccinate-release campaign plus expanded ORV efforts did not prevent the further spread of raccoon rabies. Rabies surveillance on Cape Cod became a high priority [10,11], with the aim of delineating the epizootic front to begin ORV and define priority areas for trap-vaccinate-release efforts to prevent further spread. However, in 2006, raccoon rabies was detected at the farthest tip of Cape Cod, in Provincetown. From 2006 through the spring of 2010, twice-yearly ORV treatments from Yarmouth to Provincetown were conducted using fishmeal polymer (FMP) block baits and coated sachet baits containing RABORAL V-RG^®^ vaccine (a vaccinia rabies glycoprotein recombinant oral vaccine; Merial Limited, Athens, GA, USA) distributed by hand from vehicles and on foot to reduce the enzootic area.

Cornell University began investigating the use of bait stations for the distribution of vaccine baits in New York in 2003 [12], based on a design for the control of voles (*Microtus* spp.) in orchards [13]. Experimental bait stations based partially on the Cornell design were constructed from polyvinyl chloride (PVC), filled with RABORAL V-RG FMP baits, and deployed over a 3 km^2^ study area in South Yarmouth (2006–2008) at a very high density (8/km^2^; *n* = 24) to assess their utility there. In addition to investigations into the use of bait stations by WS in Massachusetts in 2006, bait station trials also began in Florida in 2009 [14].

**Figure 2:**
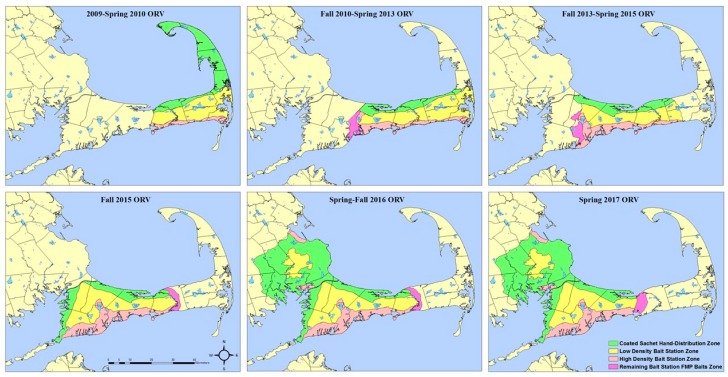
Cape Cod oral rabies vaccination baiting zones, spring 2009–spring 2017.

Due to partial funding loss reducing the CCORV program’s ability to distribute vaccine baits during 2006–2009, operational bait station use began in key locations of high human and pet densities and likely travel corridors in areas in which low raccoon densities has been indexed [15] in an attempt to treat the same area with fewer baits (Figure 2).

Five years of operational bait station use is reported as part of raccoon rabies elimination efforts on Cape Cod and compared to traditional hand-distribution of vaccine baits. In addition, since raccoon and non-target Virginia opossum (*Didelphis virginiana*) visitation to Cape Cod bait stations during 2006–2009 was statistically equivalent (1.18:1; *p* > 0.05), two bait station modifications designed to reduce opossum bait uptake were evaluated in Massachusetts and Florida. The relative performance of two vaccine bait types was also assessed to be sure these were equally useful in bait stations in Massachusetts and Florida. Lastly, the interior temperature conditions of camouflaged and unpainted white bait stations were evaluated in Massachusetts and Florida to be sure the former provided both the desired effect of preventing discovery and tampering by humans, but also to maintain adequate interior temperatures for maintaining bait and vaccine integrity.

Locations for the non-target bait uptake reduction, alternate bait trials, and interior temperature conditions in both states were selected based on habitat type. South Yarmouth, Massachusetts was selected as a representative developed coastal study location due to its mix of residential, commercial, resort, and conservation lands. The Polk County, Florida study site was selected due to its mix of wooded areas, wetlands, and urbanized or moderately-developed areas. Achieving rabies control in communities like these will play an essential role in the elimination of raccoon rabies in the U.S.

## 2. Materials and Methods

### 2.1. Bait Stations on Cape Cod

**Figure 3:**
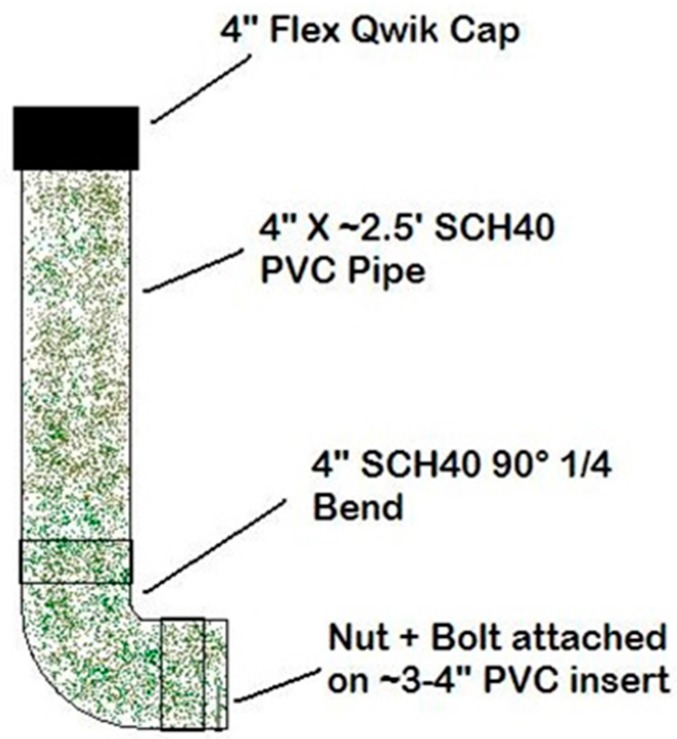
A USDA WS oral rabies vaccination standard bait station.

From 2011 through 2015, bait stations (Figure 3) were constructed and deployed operationally on Cape Cod, Massachusetts at two densities (high density—1.8 bait stations/km^2^; low density—0.1 bait stations/km^2^) based on habitat type (mixed forest, woody wetlands, or medium intensity developed, featuring relatively high raccoon densities, and pine-dominated landscapes, featuring lower raccoon densities) (Figure 2). Habitat type was determined using the National Land Cover Database (NLCD 2001). Bait stations were affixed to trees or fence posts using heavy duty cable ties, with the openings 0.3–0.5 m above the ground. RABORAL V-RG baits (*n* = 60–63) were inserted in each bait station and monitored over a three-week period in the spring (April–May) and fall (October–November (2011–2014); September–October (2015)). Bait stations were visited weekly to ensure bait stations were functioning properly and to document bait uptake. Bait location relative to bait station, bait coating, and sachet condition were recorded at each bait station. In addition to bait station use, traditional hand-distribution of baits by vehicle and foot occurred where possible with treatment type overlap avoided.

Serological surveillance occurred 4–8 weeks after each bait distribution was completed to assess rabies virus neutralizing antibody (RVNA) response in areas baited during the most recent ORV campaign. Raccoons were live captured using Tomahawk Live Trap^TM^ (Hazelhurst, WI, USA) model 108 raccoon traps baited with Hard-Core^®^ Raccoon Lure-coated marshmallows and a sardine in both bait zone types. Traps were checked every 24 h, in accordance with state law. Blood samples were taken for rabies antibody prevalence and first premolar teeth were extracted for age and tetracycline biomarker analysis. Serum samples from spring 2011–spring 2015 were sent to the Centers for Disease Control and Prevention (CDC) for analysis using the rapid fluorescent focus inhibition test (RFFIT) with a positive sero-conversion cut-off of ≥0.05 IU/mL [16]. Fisher’s exact tests were used to determine if there was a difference between seropositive raccoons within treatment zones (hand-distribution vs. bait station). Raccoons that had been previously captured and hand-vaccinated against raccoon rabies as well as juvenile raccoons that would not have been exposed to a vaccine bait were omitted from all analysis. Tetracycline biomarker is present in all RABORAL V-RG baits; however, results were not available for analysis at the time of publication. All target species were marked with ear tags that were labeled with a unique identifier to identify recaptured animals. In order to meet programmatic goals, post-ORV sampling within high-density bait station and hand-distribution zones took precedence over sampling from within low-density bait station areas.

Baits are marked with a phone number stamped on the bait matrix and on the plastic vaccine sachets to facilitate the reporting of human or pet bait contacts. Bait contact reports between bait distribution types (i.e., bait station vs. hand-distribition) were compared by Fisher’s exact tests.

### 2.2. Non-Target Bait Uptake Reduction

**Figure 4:**
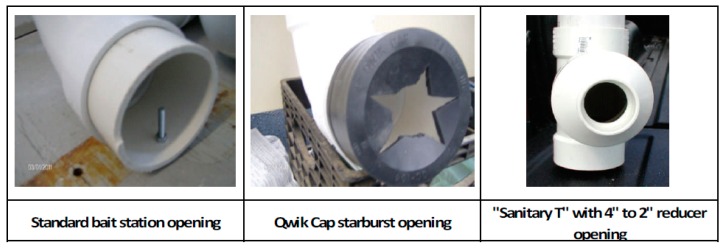
Candidate bait station modifications reducing non-target bait uptake in Barnstable County, Massachusetts and in Polk County, Florida, in 2011.

During 2011, WS initiated efforts to reduce non-target bait uptake from standard WS bait stations (Figure 3). ‘Qwik Cap Starburst’ (QCS; 4″ Fernco^TM^ (Davison, MI, USA) Qwik Cap over the opening with a star-shape cutout), and ‘Sanitary T Station’ (STS; 4″ to 2″ reducer fitting to the end) bait stations were developed. Four-inch mechanical wing nut test plugs were installed in the bottom of each STS bait station to allow for easy removal of leftover baits at the end of the trials (Figure 4).

In April 2011, two candidate bait stations types and control bait stations were deployed over 3-km^2^ study areas in Barnstable County, Massachusetts and in Polk County, Florida. Eight bait stations of each type (control, QCS, and STS) were deployed concurrently for ten nights in each state, located >0.4 km apart. Other bait station location considerations included the relative suitability of raccoon and opossum habitat, and the ability to conceal bait stations from the public. Bait station locations and types deployed were quasi-randomized (i.e., random selection from among available sites) because, in a suburban landscape, extra care had to be taken to avoid theft of the bait station and of the baits contained within. Bait stations were initially deployed with ten RABORAL V-RG baits. Bait counts were maintained in each bait station throughout the study by daily replacements of missing baits, with the exception of weekends. Automatic digital trail cameras (Massachusetts: Reconyx^TM^ RC60 (Holmen, WI, USA), Reconyx^TM^ Silent Image, and HCO^TM^ (Norcross, GA, USA); Florida: Moultrie^TM^ (Alabaster, AL, USA) GameSpy D55IR and Moultrie^TM^ I40) were used to monitor bait stations throughout the study for target and non-target visits and response to the bait stations. Bait status was recorded on a daily basis for each station. Individual animal visits were tallied, using activity breaks of 15 min as a proxy for individual identification, unless identification based on unique appearances was possible. Animal response was recorded as ‘successful’ when animals appeared to acquire a bait from a bait station, and ‘unsuccessful’ when they appeared to have been deterred. Fisher’s exact tests were used to assess whether visits to each bait station type differed between raccoons and opossums.

### 2.3. Alternate Baits for Bait Stations

ONRAB^®^ baits (adenovirus rabies glycoprotein recombinant oral vaccine in Ultralite bait matrix; Artemis Technologies, Guelph, ON, Canada) have been used in Canada and the U.S. [17,18,19]. ONRAB bait dispersal performance in bait stations was assessed relative to the more commonly used RABORAL V-RG bait. Locations for sixteen camouflaged bait stations were established in Massachusetts and Florida (8/bait type) in suitable raccoon habitats (e.g., mixed forest, wooded wetlands, or medium-intensity developed woody, wetlands) within a 3-km^2^ area based on the National Land Cover Database (NLCD 2001). Bait stations were placed at least 0.4 km apart. Locations and bait types were selected quasi-randomly as above. To mimic an actual ORV operation, 60 vaccine baits were loaded in each bait station; eight bait stations received traditional RABORAL V-RG baits, while the other eight received placebo ONRAB baits. Automatic digital trail cameras (Massachusetts: Reconyx^TM^ RC60; Florida: Moultrie^TM^ I40) were used to document wildlife activities at each bait station. Bait stations were checked daily, with the exception of weekends, from 6–16 March 2012 in Florida and 10–20 April 2012 in Massachusetts, and the number of baits remaining in each bait station was recorded. Bait condition (for those found on the ground) was recorded as: *bait matrix gone*, *bait matrix partially gone*, or *fully intact*; and *sachet punctured*, or *sachet unpunctured*. Trail camera images were analyzed daily and the number of interactions between any animal and bait or bait station was tallied. Number of target (e.g., raccoons) and non-target species (e.g., opossums, cats (*Felis catus*), dogs (*Canis lupus familiaris*)) were tallied, as well as individual actions when possible. Activity breaks of 15 min were used to separate visits from individual animals, unless unique identification of animals allowed otherwise. Fisher’s exact tests were used to assess bait consumption by each bait type.

### 2.4. Decreasing Human Interference with Bait Stations

Bait stations are generally painted with camouflage paint as a way to reduce human disturbance and theft. To determine if painting bait stations significantly increases internal temperature (which may imperil vaccine viability and bait integrity), WS constructed sixteen standard bait stations (Figure 2). Eight bait stations were used in both Massachusetts and Florida. Four bait stations from each state were painted with camouflage paint and the other four bait stations remained unpainted (white).

Paired bait stations were located in full sun, partial shade, and full shade. Locations of full sun were exposed to direct sunlight throughout the entire day. Areas of partial shade had direct sun exposure for approximately one-half of a day, or had intermittent exposure. Locations in full shade were not exposed to direct sunlight at any point. Two white bait stations and two camouflaged bait stations were deployed in locations of full sun. One bait station of each color was also deployed in the partial sun and full shade locations.

Each bait station was equipped with a maximum/minimum thermometer (Taylor^TM^ (Las Cruces, NM, USA) 5460 Indoor/Outdoor Maximum/Minimum Thermometer) and monitored on a daily basis over 10 days in Massachusetts (16–19, 23–26, and 30–31 August 2011) and 12 days in Florida (16–19, 21–26, and 28–29 August 2011). Thermometers were suspended by string at the lowest upright part of each bait station. Maximum temperatures for each bait station were recorded daily, as well as the weather conditions and any problems encountered with the thermometers. Thermometers were also deployed outside of the bait stations to document external temperatures. T-tests were used to determine differences between internal temperatures in painted and unpainted bait stations in each light exposure type.

## 3. Results

### 3.1. Bait Stations on Cape Cod

From Spring 2011–Spring 2015, adult raccoons from within the high-density bait station zone (1.8 bait stations/km^2^, 113 baits/km^2^) that had not been captured and hand-vaccinated in prior years showed seroconversion (≥0.05 IU/mL) rates of 42.1% (*n* = 126). Traditional hand-distribution of RABORAL V-RG coated sachet baits on Cape Cod (138 baits/km^2^) during the same timeframe resulted in vaccination rates of 46.2% (*n* = 182) (Table 1).

Raccoon seroconversion within high density bait station zones in Massachusetts in 2012 and 2014 resulted in higher RVNA response, however, these results were not significant (both *p* = 1.00). Seroconversion in 2011, 2013, and 2015 showed reduced seropositivity for bait stations (2011 and 2013 *p* = 1.00; 2015 *p* = 0.29) but were also not significant. Raccoons recaptured that had been previously hand-vaccinated were omitted from analysis, as well as juvenile animals that would not have encountered a bait during prior ORV operations.

In Massachusetts, areas with bait stations had significantly less bait contact calls compared to hand-baited areas from 2010–2016 (*p* < 0.001). During 2010–2016, vaccine bait distribution campaigns in Massachusetts resulted in a total of 153 bait contact calls within hand-baited areas compared with 24 from bait station-baited areas. In hand-baited areas, at least 441 baits were found by people or their pets (0.13%). However, within areas baited by bait stations, only 38 baits were involved with a pet or human bait contact (0.02%).

### 3.2. Non-Target Bait Uptake Reduction

During April 2011 in Massachusetts and Florida, raccoons accounted for 74.8% of the apparent successful visits to control bait stations, while 25.2% were by opossums (*n* = 107). A similar relationship was observed for QCS bait stations, where 82.6% of apparent successful visits to bait stations were by raccoons and 17.4% were by opossums (*n* = 138). However, the ratio of raccoons photographed at STS bait stations was higher than the control and QCS bait stations (90.9%) and lower for opossums (9.1%; *n* = 44).

In Massachusetts, visitation and apparent bait retrieval by raccoons and opossums significantly differed by opening type (*p* < 0.001). Control bait stations visited in Massachusetts had a 1.8:1 raccoon to opossum ratio, while QCS and STS bait stations had raccoon to opossum visitation ratios of 55:1 and 14:1, respectively. However, apparent access by raccoons and opossums in Florida did not differ (*p* = 0.131).

### 3.3. Alternate Baits for Bait Stations

In Massachusetts, all bait stations containing ONRAB baits were emptied and all baits were consumed and/or punctured. Only one bait station containing RABORAL V-RG baits was completely emptied of baits by the end of the study. Of the 240 RABORAL V-RG baits originally deployed, 122 remained on day 10. In Florida, all baits were taken from the bait stations by the end of the study; however, one bait station containing ONRAB baits in Florida had baits through day 9, but was emptied prior to the day 10 check. In Massachusetts, all intact baits that had been pulled out of the bait station to the ground were redeployed in each bait station to maximize documenting bait-uptake and bait station access. Baits on the ground in Florida were left in front of the bait station to document if they would be consumed after the bait station was emptied. Of the baits left on the ground at the bait stations in Florida, all were consumed by day 6. The relation between the removal of ONRAB and RABORAL V-RG baits was statistically significant (*p* < 0.001) in Massachusetts, but not in Florida (*p* = 1.00).

### 3.4. Decreasing Human Interference with Bait Stations

**Figure 5:**
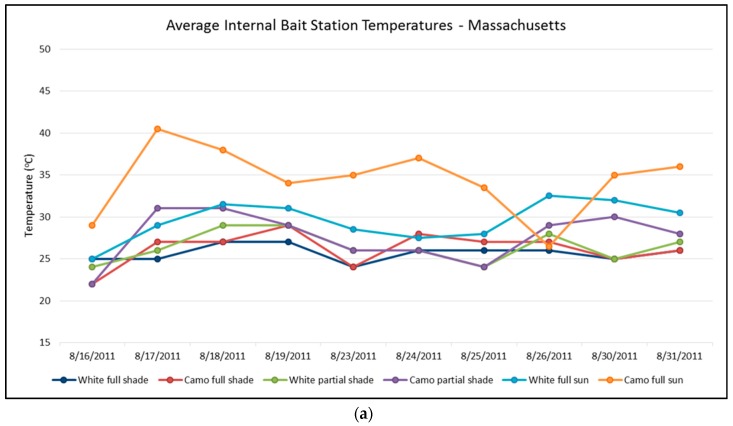

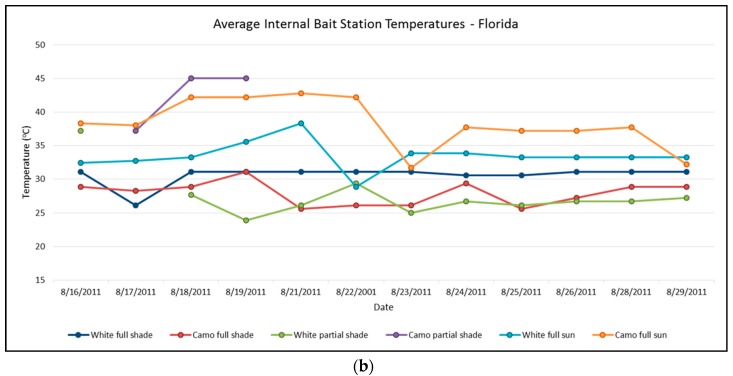
(**a**) Average internal bait station temperatures in Massachusetts. (**b**) Average internal bait station temperatures in Florida.

In Massachusetts, as might be expected, bait stations deployed in areas of full sun had a higher average temperature than those in shady locations. Camouflaged bait stations were also warmer than white bait stations, especially in full sun locations (*p* = 0.009), averaging 35.5 °C compared to 29.6 °C. Both camouflaged and white bait stations in full shade locations had a similar average high temperature of 26.2 °C and 25.7 °C, respectively (*p* = 0.32). Similarly, camouflaged and white bait stations also had similar average high temperatures in partial shade areas (27.6 °C and 26.4 °C, respectively) (*p* = 0.13). Several camouflaged bait stations in full sun showed high temperatures of over 40 °C (Figure 5a).

In Florida, camouflaged bait stations in partial shade had higher average temperatures (42.8 °C) than any of the other bait stations. In full sun, camouflaged bait stations were warmer (38.6 °C), but they also had a slightly cooler average low temperature than white bait stations (24.4 °C and 25.3 °C, respectively). In full shade, white bait stations were warmer than camouflaged bait stations (30.6 °C and 27.9 °C, respectively) while both remaining about the same temperature at night (24.0 °C and 24.2 °C, respectively). Average interior temperatures for camouflaged and white bait stations in Florida were statistically different in full shade, partial shade, and full sun (*p* = 0.0003, 0.017, & 0.001, respectively). In each location, average internal temperatures within white bait stations were significantly cooler than camouflage bait stations (Figure 5b).

## 4. Discussion

### 4.1. Bait Stations on Cape Cod

When WS lost cooperative program funding for the CCORV in 2009, the use of bait stations was a logical next step, given that they could be efficiently deployed in more developed areas, and to intercept raccoons moving through apparently poorer raccoon habitats where broadcast baiting is likely wasteful [15]. As the ORV zone shifted west due to decreased raccoon rabies cases in treatment areas, baiting was increasingly conducted in more highly developed areas, increasing the need to switch to bait stations to reduce potential bait contacts. The use of bait stations on Cape Cod resulted in similar RVNA responses to those areas treated by hand-distribution of baits. This suggests that bait stations hold promise for ORV in the more highly-developed areas to be encountered as the NRMP approaches elimination of raccoon rabies along the eastern seaboard [20].

Bait contact by people or pets is a concern and therefore, reduction in bait contacts is desired. Additionally, every discovered bait potentially represents a lost dose for an unvaccinated raccoon. As evidenced by the reduced number of bait contact-related phone calls and reports, the use of bait stations in developed areas has resulted in fewer contact events. The use of bait stations will continue to play an important role in reducing these bait contacts, especially in heavily developed areas.

### 4.2. Non-Target Bait Uptake Reduction

During a previous assessment of bait station visitation by non-target species on Cape Cod, raccoon visits to bait stations occurred at a similar frequency to non-target opossums (1.18:1; *p* > 0.05). Based on the low total number of apparent successful raccoon and opossum visits to STS bait stations compared to visits to control and QCS bait stations, it appears that this bait station prohibits easy access to baits by both species. Consequently, STS bait stations are not suitable for distribution of vaccine baits. Results of the bait station opening study did not differ between tested modifications in Florida. However, in Massachusetts, raccoon to opossum successful visitation ratios improved at QCS and STS bait stations, although the number of raccoon visits to STS bait stations decreased. Consequently, the strategy of modifying bait station openings seems to hold promise for reducing the waste represented by non-target bait consumption, and further trials will likely be conducted with better photographic equipment to more clearly document bait uptake. The control bait station design remains the best operational design until further trails are conducted.

A number of unquantifiable factors could account for differences between Massachusetts and Florida results, such as raccoon and opossum population density differences, weather, and food item competition in the surrounding landscape [21]. An obvious limitation in this assessment was the lack of complete certainty that a visit resulted in actual bait access or uptake. Future use of increasingly available video options to capture more definitive evidence that animals visiting bait stations are actually obtaining baits may help clarify this. In addition to camera capture data, serologic data would be important when comparing bait uptake from different bait station opening modifications, which would require increased geographic areas and effort.

Skunks found within the Massachusetts and Florida study locations (striped skunks, *Mephitis mephitis* (MA and FL) and spotted skunks, *Spilogale putorius* (FL)) were not documented visiting any bait stations during this study, but their roles as potential bait competitors need further attention based on their relative densities in many locations suitable for bait station use.

### 4.3. Alternate Baits for Bait Stations

In Massachusetts, ONRAB baits performed better in bait stations than traditional RABORAL V-RG baits based on successful removal. Both baits performed equally as well in Florida. Given the mixed results for bait removal by type between the two locations, further testing is necessary to determine whether real performance differences exist. In addition, since bait stations often dispense baits over a two- to three-week period, future bait station bait performance assessments should occur under conditions where vaccine titers can be assessed. Despite ONRAB baits preference by raccoons on Cape Cod, RABORAL V-RG baits continue to be used, and will be until further examination of ONRAB baits in bait stations is conducted.

Competition for baits, primarily in areas with high non-target populations, limits consumption by target raccoons. In Massachusetts, raccoons were photographed at bait stations containing ONRAB baits more frequently than at bait stations containing RABORAL V-RG baits, while bait stations containing RABORAL-VRG baits were more frequented by opossums. It did not appear that raccoons or opossums showed any preference towards either bait type in Florida. Further study may be necessary to examine bait-type preference on a location-by-location basis, as preference may vary by geographic region. Additional bait station modification may be necessary to prevent non-target uptake in both locations.

### 4.4. Decreasing Human Interference with Bait Stations

Human discovery of bait stations is problematic due to tampering, theft and increased potential for human vaccine contact. However, before bait stations could be uniformly camouflaged, it was important to assess whether these modifications could affect bait and vaccine quality. The most obvious candidate agent for reduced quality was assumed to be internal temperature. As a result of this study, it is recommended that bait stations be deployed in areas of full shade, as internal temperatures in those bait stations were cooler, regardless of color. Operationally, bait stations are rarely placed in areas of full sun due to visibility by the public and to prevent the excessive heating of vaccine baits. Where it became absolutely necessary in some instances to place bait stations in areas of full sun, white bait stations would be recommended rather than camouflage bait stations as a result of this study. Differing results between Massachusetts and Florida may be accounted for by higher ambient temperatures in Florida. However, given the need for vaccine and bait stability, these trials might bear repeating under more controlled circumstances. Additionally, bait and vaccine thermostability trials need to be conducted to examine efficacy of the vaccine at varying times throughout the typical three-week timeframe in which bait stations may contain baits.

## 5. Conclusions

Integrating bait stations into rabies control efforts on Cape Cod has led to similar sero-conversion rates and wildlife rabies management performance compared to traditional hand-baiting alone. Additionally, the use of bait stations has reduced bait contact reports, maximizing the number of baits available to raccoons and other target species. Alternate bait station openings may eventually play a role in limiting non-target bait uptake. Furthermore, it was discovered that ONRAB vaccine baits were removed at a faster rate by target and non-target species when compared to RABORAL V-RG. This study also showed that bait stations should be placed in areas with limited sun exposure when possible. Although serology results are an important component to the evaluation of ORV success, the ultimate factors are the absence of raccoon rabies cases in the currently-treated zone, and those areas from where ORV has been shifted away. On Cape Cod from 2014–2016, 542 animals were tested for rabies, of which six were confirmed positive for raccoon rabies. All six animals (two raccoons and four skunks) were from west of the Cape Cod Canal. This integrated approach, subject to continued trials to achieve better bait station performance, may play an important role in rabies control and prevention in other areas of the U.S., especially as rabies management turns its focus from preventing raccoon rabies spread to elimination from the eastern U.S. However, some important questions regarding optimal bait station design and effectiveness remain.

## Figures and Tables

**Table 1:** Post-ORV raccoon serology rates on Cape Cod, MA—2011–2015.

Year	Percent Seropositive by Hand Distribution (*n*; 95% CI) (138 Baits/Km^2^)	Percent Seropositive by High Density Bait Station (*n*; 95% CI) (1.8 Bait Stations/Km^2^, 113 Baits/Km^2^)
2011	43.9 (41; 0.30, 0.59)	38.5 (13; 0.18, 0.64)
2012	38.5 (13; 0.18, 0.64)	41.1 (17; 0.22, 0.64)
2013	36.1 (36; 0.22, 0.52)	31.6 (19; 0.15, 0.54)
2014	38.5 (39; 0.25, 0.54)	40.5 (37; 0.26, 0.57)
2015	62.3 (53; 0.49, 0.74)	50.0 (40; 0.35, 0.65)
Total (2011–2015)	46.2 (182; 0.39, 0.53)	42.1 (126; 0.34, 0.51)

## References

[1] Slate D., Algeo T.P., Nelson K.M., Chipman R.B., Donovan D., Blanton J.D., Niezgoda M., Rupprecht C.E. (2009). Oral rabies vaccination in North America: Opportunities, complexities, and challenges. PLoS Negl. Trop. Dis..

[2] Blancou J. (2008). The control of rabies in Eurasia: Overview, history and background. Dev. Biol..

[3] Wandeler A.I. (2008). The rabies situation in western Europe. Dev. Biol..

[4] MacInnes C.D., Smith S.M., Tinline R.R., Ayers N.R., Bachmann P., Ball D.G., Calder L.A., Crosgrey S.J., Fielding C., Hauschildt P. (2001). Elimination of rabies from red foxes in eastern Ontario. J. Wildl. Dis..

[5] Rosatte R.C., Power M.J., Donovan D., Davies J.C., Allan M., Bachmann P., Stevenson B., Wandeler A., Muldoon F. (2007). Elimination of arctic variant rabies in red foxes, metropolitan Toronto. Emerg. Infect. Dis..

[6] Rosatte R.C., Donovan D., Allan M., Bruce L., Buchanan T., Sobey K., Stevenson B., Gibson M., MacDonald T., Whalen M. (2009). The control of raccoon rabies in Ontario Canada: Proactive and reactive tactics, 1994–2007. J. Wildl. Dis..

[7] Fearneyhough M.G., Wilson P.J., Clark K.A., Smith D.R., Johnston D.H., Hicks B.N., Moore G.M. (1998). Results of an oral rabies vaccination program for coyotes. J. Am. Vet. Med. Assoc..

[8] Velasco-Villa A., Reeder S.A., Orciari L.A., Yager P.A., Franka R., Blanton J.D., Zuckero L., Hunt P., Oertli E.H., Robinson L.E. (2008). Enzootic rabies elimination from dogs and reemergence in wild terrestrial carnivores, United States. Emerg. Infect. Dis..

[9] Sidwa T.J., Wilson P.J., Moore G.M., Oertli E.H., Hicks B.N., Rohde R.E., Johnston D.H. (2005). Evaluation of oral rabies vaccination programs for control of rabies epizootics in coyotes and gray foxes: 1995–2003. J. Am. Vet. Med. Assoc..

[10] Bjorklund B.M., Algeo T.P., Chandler M.D., Wilda D.J., Slate D., Timm R., O’Brien J. (2006). Terrestrial rabies surveillance on Cape Cod: A community-based multi-agency strategy to provide critical information for rabies control. Proceedings of the 22nd Vertebrate Pest Conference.

[11] Algeo T.P., Chipman R.B., Bjorklund B.M., Chandler M.D., Wang X., Slate D., Rupprecht C.E. (2008). Anatomy of the Cape Cod oral rabies vaccination program. Proceedings of the 23rd Vertebrate Pest Conference.

[12] Boulanger J.R., Bigler L.L., Curtis P.D., Lein D.H., Arthur J. (2006). A polyvinyl chloride bait station for dispensing rabies vaccine to raccoons in suburban landscapes. Wildl. Soc. Bull..

[13] Tobin M.E., Richmond M.E. Bait Stations for Controlling Voles in Apple Orchards. Proceedings of the Third Eastern Wildlife Damage Control Conference.

[14] Haley B.S., Algeo T.P., Bjorklund B., Duffiney A.G., Hartin R.E., Martin A., Nelson K.M., Chipman R.B., Slate D. (2017). Evaluation of bait station density for oral rabies vaccination of raccoons in urban and rural habitats in Florida. Trop. Med. Infect. Dis..

[15] Algeo T., Slate D., Caron R., Atwood T., Recuenco S., Ducey M., Chipman R., Palace M. (2017). Modeling raccoon (*Procyon lotor*) habitat connectivity to identify potential corridors for rabies spread. Trop. Med. Infect. Dis..

[16] Smith J.S., Yager P.A., Baer G.M., Meslin F.X., Kaplan M.M., Koprowski H. (1996). A rapid fluorescent focus inhibition test (RFFIT) for determining rabies virus-neutralizing antibody. Laboratory Techniques in Rabies.

[17] Fehlner-Gardiner C., Rudd R., Donovan D., Slate D., Kempf L., Badcock J. (2012). Comparing ONRAB^®^ and RABORAL V-RG^®^ oral rabies vaccine field performance in raccoons and striped skunks, New Brunswick, Canada, and Maine, USA. J. Wildl. Dis..

[18] Slate D., Chipman R.B., Algeo T.P., Mills S.A., Nelson K.M., Croson C.K., Dubovi E.J., Vercauteren K., Renshaw R.W., Atwood T. (2014). Safety and immunogenicity of Ontario rabies vaccine bait (ONRAB) in the first US field trial in raccoons (*Procyon lotor*). J. Wildl. Dis..

[19] Rosatte R.C., Donovan D., Davies J.C., Allan M., Bachmann P., Stevenson B., Sobey K., Brown L., Silver A., Bennett K. (2009). Aerial distribution of onrab baits as a tactic to control rabies in raccoons and striped skunks in Ontario, Canada. J. Wildl. Dis..

[20] Anderson A., Shwiff S.A., Chipman R.B., Atwood T., Cozzens T., Fillo F., Hale R., Hatch B., Maki J., Rhodes O.E. (2014). Forecasting the spread of raccoon rabies using a purpose-specific group decision-making process. Hum. Wildl. Interact..

[21] Bjorklund B.M., Thomas H.H., Palmiotto P.A., Algeo T.P., Slate D., Chipman R.B., Chandler M.D., Wilda D.J., Timm R.M., Madon M.B. Potential food item distractions during raccoon ORV baiting campaigns on Cape Cod, Massachusetts: would you like fries with that?. Proceedings of the 23rd Vertebrate Pest Conference.

